# Supplementary material for: Characterizations and Antibacterial Efficacy of Chitosan Oligomers Synthesized by Microwave-Assisted Hydrogen Peroxide Oxidative Depolymerization Method for Infectious Wound Applications
Source: Materials (Basel). 2021 Aug 10;14(16):4475. doi: 10.3390/ma14164475 (PMC8399586; doi:10.3390/ma14164475)
Supplement: Supplementary file 1 [file materials-14-04475-s001.zip › materials-1289456-supplementary.pdf]

Supplementary Materials

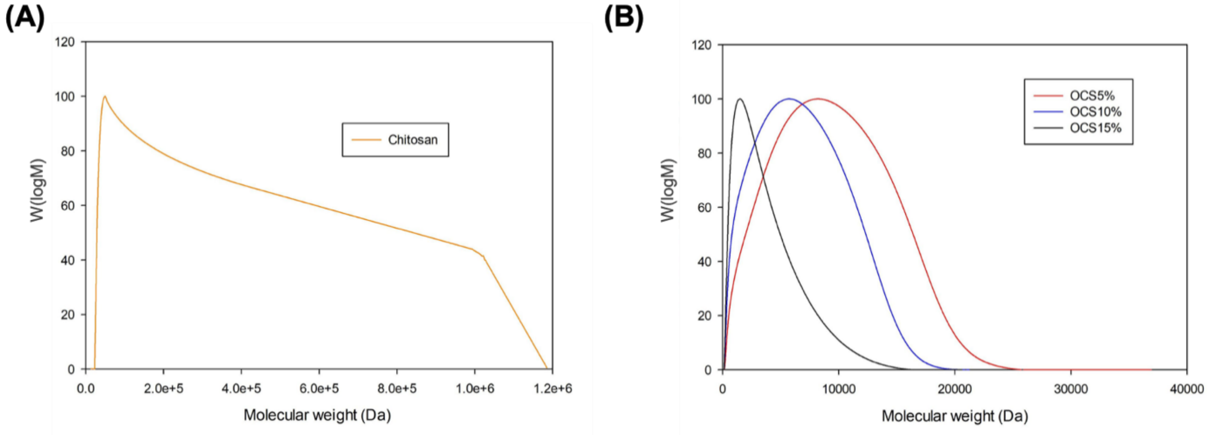

Figure S1. Molecular weight distribution of (**A**) chitosan and (**B**) OCS samples.
